# Detection characteristics and antimicrobial resistance profiles of *Salmonella* spp. isolated from fecal cultures of inpatients at a maternal and child healthcare hospital in Shenzhen: a 5-year retrospective study

**DOI:** 10.1128/spectrum.01536-26

**Published:** 2026-10-08

**Authors:** Tongyan Ding, Shaoxiang Lin, Xiaochun Liu, Shujuan Yang, Futing Liao, Qiaoxin Zhang, Zhenwen Zhou

**Affiliations:** 1 Clinical Laboratory, Key Laboratory of Bacterial Antimicrobial Resistance and Prevention, Medical Research Institute of Maternal and Child, Longgang District Maternity & Child Healthcare Hospital of Shenzhen City (Affiliated Shenzhen Women and Children's Hospital (Longgang) of Shantou University Medical College) 85113, Shenzhen, Guangdong, China; 2 The First Affiliated Hospital of Shantou University Medical College 485866, Shantou, Guangdong, China; 3 Clinical Laboratory, Guangzhou Women and Children's Medical Center, Guangzhou Medical University, Guangdong Provincial Clinical Research Center for Laboratory Medicine 26468, Guangzhou, Guangdong, China; Newcastle University, Newcastle upon Tyne, United Kingdom

**Keywords:** *Salmonella *spp., fecal culture, antimicrobial resistance, maternal and child healthcare hospital, Shenzhen

## Abstract

*Salmonella* spp. remain an important cause of bacterial gastrointestinal infection, but local data from maternal and child healthcare hospitals remain limited. This retrospective study analyzed *Salmonella* spp. isolated from fecal cultures of inpatients at Longgang District Maternity and Child Healthcare Hospital of Shenzhen City from 2021 to 2025. Fecal specimens were processed using routine selective culture procedures; isolates were identified with the VITEK 2 Compact system, with VITEK MS confirmation when required, and antimicrobial susceptibility was interpreted according to Clinical and Laboratory Standards Institute (CLSI) M100, 35th edition. Annual positivity and non-duplicate isolation rates were compared using Pearson χ^2^ tests. A total of 106 non-duplicate isolates were recovered, including 98 from pediatric inpatients. Annual positivity and non-duplicate isolation rates differed significantly across the 5-year study period (both *P* < 0.001) and were highest in 2025. Among pediatric cases, children aged 1–3 years accounted for 79.6%, with the 12 to <24-month group accounting for 52.0%. Resistance was high to ampicillin (76.7%), ampicillin/sulbactam (63.3%), trimethoprim-sulfamethoxazole (46.6%), and ceftriaxone (36.9%). Resistance to ciprofloxacin and levofloxacin was 12.5% and 14.8%, respectively, although intermediate rates were high. Most isolates remained susceptible to piperacillin/tazobactam and carbapenems. These findings show a concentration of laboratory-detected *Salmonella* among children aged 1–3 years, particularly those aged 12 to <24 months, and substantial resistance to several commonly used agents, supporting continued local surveillance and susceptibility-guided antimicrobial use.

**IMPORTANCE:**

*Salmonella* spp. remain an important bacterial cause of gastrointestinal infection in children, but local data from maternal and child healthcare hospitals are limited. This 5-year retrospective study describes the detection characteristics and antimicrobial resistance profiles of *Salmonella* spp. isolated from fecal cultures of inpatients in a maternal and child healthcare hospital in Shenzhen, China. The findings show that *Salmonella* isolates were mainly recovered from young pediatric patients and exhibited high resistance to several commonly used antimicrobial agents, including ampicillin, ampicillin/sulbactam, trimethoprim-sulfamethoxazole, and ceftriaxone, while reduced susceptibility to fluoroquinolones was also observed. These results provide institution-specific evidence for empirical treatment decisions, antimicrobial stewardship, and continued surveillance of enteric bacterial pathogens in pediatric and maternal-child healthcare settings.

## INTRODUCTION

Acute gastrointestinal infections are among the most common infectious diseases worldwide and occur frequently in infants, pregnant women, and individuals with relatively vulnerable immune status (1–4). Maternal and child healthcare hospitals mainly serve neonates, children, and pregnant women. These populations either have immature immune systems or are in special physiological states, making them more susceptible to enteric pathogens and potentially more prone to rapid disease progression after infection (5). Global burden studies have shown that diarrheal diseases remain an important cause of morbidity and mortality among children under 5 years of age (6). Although overall mortality has declined in recent years, children continue to bear a substantial proportion of the disease burden. Stool culture remains a fundamental laboratory method for the diagnosis of bacterial enteric infections and plays an important role in etiological confirmation, targeted antimicrobial therapy, and surveillance of regional pathogen trends (7).

In recent years, the widespread use of antimicrobial agents and persistent selection pressure have contributed to the growing problem of antimicrobial resistance among enteric pathogens, making it a major global public health concern (8–10). Among bacterial enteric pathogens, *Salmonella* spp. are an important cause of diarrhea, fever, and foodborne gastroenteritis, and they are of particular concern in children, pregnant women, and other priority populations (11, 12). Previous studies have reported increasing resistance of *Salmonella* spp. to ampicillin, fluoroquinolones, and some cephalosporins in certain regions. The emergence of multidrug-resistant strains further complicates empirical antimicrobial therapy (13–15). For maternal and child healthcare populations, antimicrobial selection must consider not only pathogen coverage but also gestational age, placental transfer, and potential fetal or neonatal effects in pregnant patients, as well as age- and weight-based dosing, developmental pharmacology, and class-specific precautions or restrictions in children (16). National surveillance further indicates that the temporal and regional features of human salmonellosis outbreaks vary across China (17). Therefore, timely understanding of the distribution and resistance profiles of common enteric pathogens, represented by *Salmonella* spp., has important clinical relevance.

Shenzhen is a rapidly growing megacity with a large migrant population, a comparatively young age structure, and high residential mobility (18–20). Longgang District also receives patients who move across districts or regions for maternal and pediatric care. These demographic and healthcare-seeking characteristics create a large and heterogeneous service population and may influence exposure patterns, testing volume, and the distribution of enteric pathogens observed in hospital-based surveillance. As a common enteric pathogen of concern in children and pregnant women, the prevalence and resistance characteristics of *Salmonella* spp. may vary by region, healthcare institution type, and patient population (21, 22). Local data on the detection and antimicrobial resistance of *Salmonella* spp. in maternal and child healthcare hospitals are therefore important for optimizing empirical therapy, strengthening antimicrobial stewardship, and improving infection prevention and control.

However, long-term continuous surveillance data on *Salmonella* spp. isolated from fecal cultures of inpatients in maternal and child healthcare institutions remain limited, especially with respect to population distribution and antimicrobial resistance based on local hospital data. Therefore, this study retrospectively analyzed *Salmonella* spp. isolated from fecal cultures of inpatients at a maternal and child healthcare hospital in Shenzhen from 2021 to 2025. We focused on annual detection patterns, population distribution, and antimicrobial resistance profiles, with the aim of providing local evidence for precise diagnosis and treatment of gastrointestinal infections and rational antimicrobial use among maternal, infant, and pediatric populations in Shenzhen, as well as supporting the development of prevention and control strategies in maternal and child healthcare settings.

## MATERIALS AND METHODS

### Study design and isolate source

This was a single-center retrospective observational study. Data were obtained from the laboratory information system of Longgang District Maternity and Child Healthcare Hospital of Shenzhen City. The study period was from 1 January 2021 to 31 December 2025. The study included *Salmonella* spp. isolates recovered from fecal specimens submitted by inpatients during this period. A total of 106 eligible non-duplicate *Salmonella* spp. isolates were included. All clinical specimens included in the analysis were fecal specimens. This study was based on routine clinical laboratory data and used only de-identified laboratory results. It did not involve direct patient contact, additional sample collection, or clinical intervention.

### Definitions and deduplication

Records were linked using the hospital patient identifier stored in the laboratory information system. If a patient had more than one *Salmonella*-positive fecal culture during the entire study period, only the earliest isolate was retained, irrespective of the interval between cultures or any difference in antimicrobial susceptibility profile. Deduplication was therefore patient based; whole-genome sequencing, targeted sequencing, and other molecular typing methods were not used to determine whether isolates were genetically identical. The annual positivity rate was defined as the number of *Salmonella*-positive fecal specimens divided by the number of fecal specimens submitted in that year. The annual non-duplicate isolation rate was defined as the number of included non-duplicate isolates divided by the number of fecal specimens submitted in that year. Patient age was calculated from date of birth to specimen-collection date and categorized by completed year of age. Accordingly, the 1-year age group comprised patients aged at least 12 months but younger than 24 months.

### Isolation, identification, and antimicrobial susceptibility testing

Fecal specimens were processed according to the routine stool-culture standard operating procedure of the clinical microbiology laboratory. Specimens were inoculated onto selective and differential enteric culture media, with enrichment performed when indicated by the routine protocol, and incubated aerobically at 35–37°C for 18–48 h. Colonies with morphology suggestive of *Salmonella* were subcultured to obtain pure growth before identification and susceptibility testing. Preliminary bacterial identification and antimicrobial susceptibility testing were performed using the VITEK 2 Compact automated microbial identification system (bioMérieux, France). For isolates that could not be reliably identified by the automated system or for which the identification results were uncertain, further confirmation was performed using the VITEK MS matrix-assisted laser desorption ionization–time of flight mass spectrometry system (MALDI-TOF MS; bioMérieux, France). Antimicrobial susceptibility results were interpreted according to the Clinical and Laboratory Standards Institute (CLSI) Performance Standards for Antimicrobial Susceptibility Testing, M100, 35th edition, 2025.

### Quality control

Standard quality control strains were used for performance verification during testing. *Escherichia coli* ATCC 25922 was used as the routine quality control strain. All quality control results were within the acceptable ranges specified by CLSI M100, 35th edition, 2025.

### Data collection and statistical analysis

Relevant information on included *Salmonella* isolates was extracted from the laboratory information system. Descriptive statistics were used for data organization and analysis. Categorical variables were expressed as counts and percentages. The main analyses included the detection characteristics of *Salmonella* spp. from fecal specimens of inpatients from 2021 to 2025 and their antimicrobial resistance profiles to commonly tested antimicrobial agents. Pearson χ^2^ tests were used to compare the annual positivity rates and annual non-duplicate isolation rates across the 5-year study period. Age and sex distributions were summarized descriptively because age- and sex-specific denominators for all submitted specimens were unavailable. All tests were two-sided, and *P* < 0.05 was considered statistically significant. These tests evaluated proportions among specimens submitted to the hospital laboratory and were not interpreted as estimates of population-level incidence.

## RESULTS

### Detection of *Salmonella* spp. among fecal culture isolates

From 2021 to 2025, a total of 4,201 fecal specimens were submitted by inpatients, of which 176 were positive for *Salmonella* spp., corresponding to an overall positivity rate of 4.2%. After duplicate removal, 106 non-duplicate *Salmonella* spp. isolates were included for subsequent analysis. The numbers of fecal specimens submitted each year from 2021 to 2025 were 927, 860, 798, 787, and 829, respectively. The corresponding numbers of *Salmonella*-positive specimens were 23, 17, 28, 28, and 80, with positivity rates of 2.5%, 2.0%, 3.5%, 3.6%, and 9.7%, respectively. The numbers of non-duplicate *Salmonella* isolates were 15, 14, 16, 16, and 45, corresponding to non-duplicate isolation rates of 1.6%, 1.6%, 2.0%, 2.0%, and 5.4%, respectively. The annual positivity rate differed significantly across the 5 years (Pearson χ^2^ = 80.52, df = 4, *P* < 0.001), as did the annual non-duplicate isolation rate (Pearson χ^2^ = 35.97, df = 4, *P* < 0.001). Both rates were highest in 2025 (Table 1).

**TABLE 1:** Number of fecal specimens submitted by inpatients, *Salmonella*-positive specimens, and non-duplicate *Salmonella* isolates from 2021 to 2025^*a*^

Year	No. of specimens submitted	No. of *Salmonella*-positive specimens	Positivity rate (%)	No. of non-duplicate *Salmonella* isolates	Non-duplicate isolation rate (%)
2021	927	23	2.5	15	1.6
2022	860	17	2.0	14	1.6
2023	798	28	3.5	16	2.0
2024	787	28	3.6	16	2.0
2025	829	80	9.7	45	5.4
Total	4,201	176	4.2	106	2.5

^*a*^ Rates were calculated per submitted fecal specimens. Both annual positivity and non-duplicate isolation rates differed across years (*P* < 0.001). Deduplication retained each patient’s earliest isolate; no sequencing was used.

### Distribution of *Salmonella* isolates in pediatric patients and by sex

**Fig 1:**
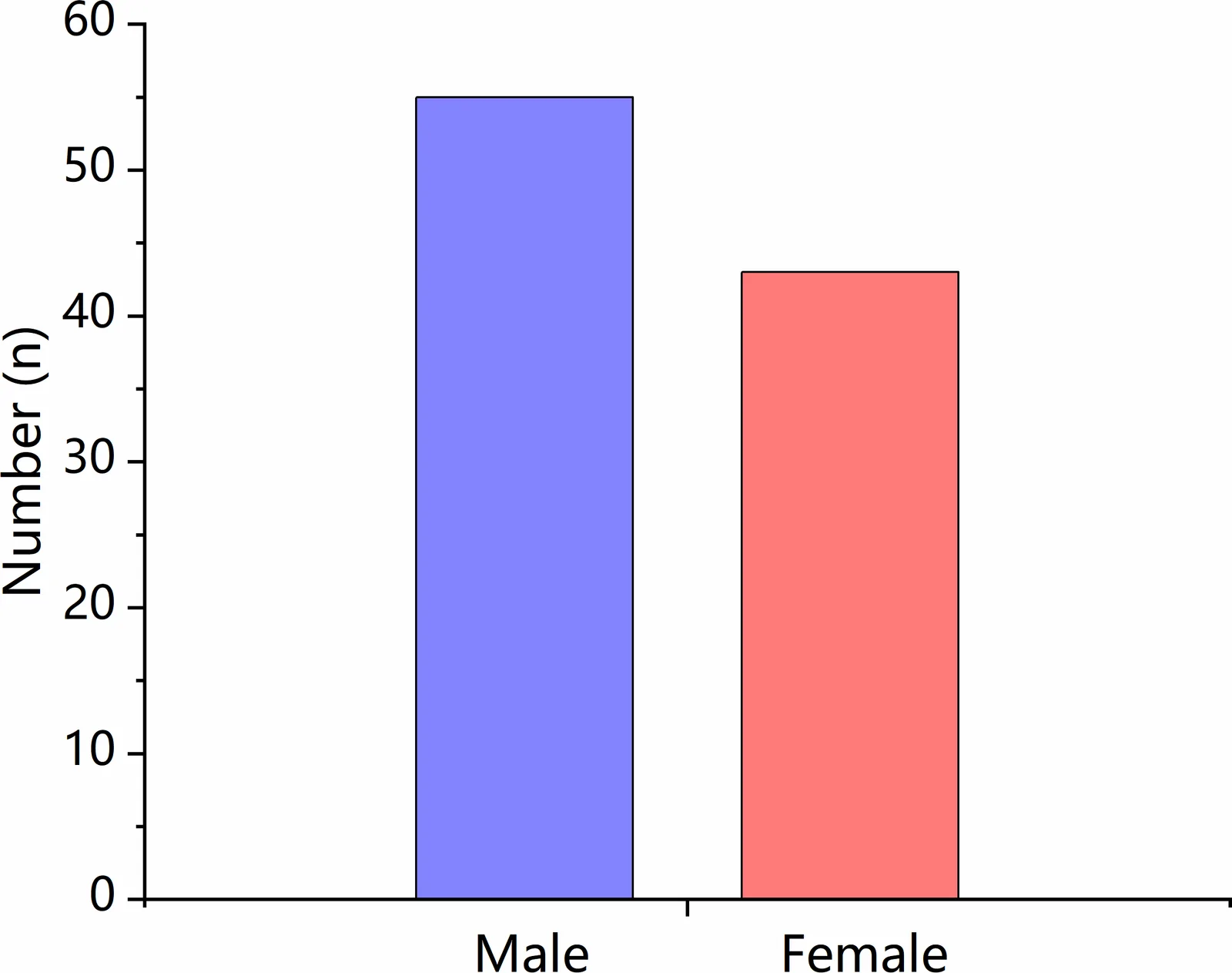
Sex distribution of pediatric patients from whom *Salmonella* spp. were isolated.

As shown in Fig. 1, among the 106 non-duplicate *Salmonella* spp. isolates included from 2021 to 2025, 98 were recovered from pediatric inpatients, while the remaining 8 were from non-pediatric inpatients and were not further stratified because of the small sample size. Among the 98 non-duplicate *Salmonella* isolates from pediatric inpatients, 55 were from male patients and 43 were from female patients, accounting for 56.1% and 43.9%, respectively. The proportion of isolates from male patients was slightly higher than that from female patients, with a difference of 12.2 percentage points and a male-to-female ratio of approximately 1.28:1. Overall, *Salmonella* isolates from pediatric inpatients showed a mild male predominance in sex distribution.

### Age distribution of *Salmonella* isolates

**Fig 2:**
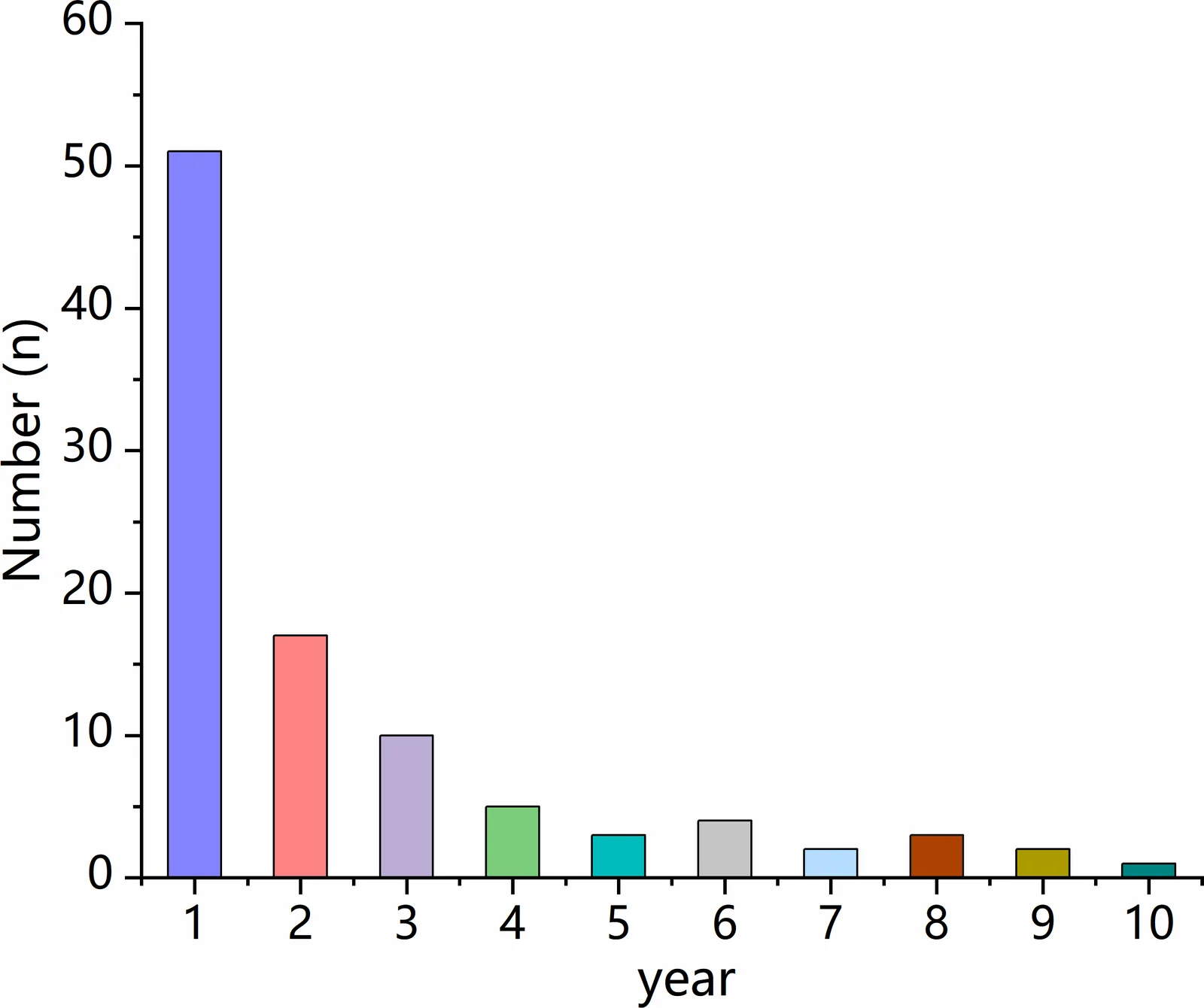
Age distribution of *Salmonella* infections in pediatric patients.

As shown in Fig. 2, pediatric cases of *Salmonella* infection were concentrated mainly in younger age groups. Children aged 1 year accounted for the largest number of cases, with 51 cases, representing 52.0% of all pediatric cases. The 1-year age group was defined as 12 to <24 months; no pediatric case was recorded in the <12-month (age 0) category. Children aged 1–3 years accounted for 78/98 pediatric cases (79.6%). The number of cases generally decreased with increasing age. There were 17 cases among children aged 2 years (17.3%) and 10 cases among those aged 3 years (10.2%). The number of cases was markedly lower among children aged 4 years and older, with five cases at 4 years (5.1%), three cases at 5 years (3.1%), and four cases at 6 years (4.1%). Cases were less frequent among children aged 7–10 years, with 2 (2.0%), 3 (3.1%), 2 (2.0%), and 1 (1.0%) cases, respectively. Overall, *Salmonella* detection was mainly observed in children aged 1 year and other younger pediatric age groups, with detection numbers gradually decreasing as age increased.

### Antimicrobial resistance profile of *Salmonella* spp.

As shown in Table 2, the resistance levels of *Salmonella* spp. varied markedly across different classes of antimicrobial agents. The highest resistance rate was observed for ampicillin (76.7%). Resistance rates to ampicillin/sulbactam and trimethoprim-sulfamethoxazole were also relatively high, at 63.3% and 46.6%, respectively. Among cephalosporins, resistance rates to ceftriaxone and ceftazidime were 36.9% and 22.3%, respectively, whereas the resistance rate to cefepime was comparatively lower at 13.8%. *β*-lactam/*β*-lactamase inhibitor combinations generally retained good *in vitro* activity, with a low resistance rate to piperacillin/tazobactam of 3.0%. Carbapenems also maintained high activity, with susceptibility rates of 97.1% for imipenem and 98.1% for ertapenem. In addition, susceptibility rates to aztreonam and amoxicillin/clavulanic acid were relatively high, at 90.0% and 86.3%, respectively. Tigecycline and nitrofurantoin also showed favorable *in vitro* activity against *Salmonella* spp., with susceptibility rates of 94.5% and 100.0%, respectively. Among fluoroquinolones, resistance rates to ciprofloxacin and levofloxacin were 12.5% and 14.8%, respectively, but the intermediate rates were high, reaching 50.0% and 64.8%, respectively.

**TABLE 2:** Antimicrobial resistance profile of *Salmonella* spp. to commonly used antibiotics^*a*^

Antimicrobial agents	Resistance rate (%)	Susceptibility rate (%)	Intermediate rate (%)
Ampicillin	76.7	23.3	0.0
Ampicillin/sulbactam	63.3	30.0	6.7
Piperacillin/tazobactam	3.0	97.0	0.0
Ceftriaxone	36.9	63.1	0.0
Ceftazidime	22.3	75.7	1.9
Cefepime	13.8	86.2	0.0
Aztreonam	10.0	90.0	0.0
Imipenem	1.9	97.1	1.0
Ertapenem	1.9	98.1	0.0
Ciprofloxacin	12.5	37.5	50.0
Levofloxacin	14.8	20.5	64.8
Amoxicillin/clavulanic acid	5.5	86.3	8.2
Trimethoprim/sulfamethoxazole	46.6	53.4	0.0
Tigecycline	1.4	94.5	4.1
Nitrofurantoin	0.0	100.0	0.0

^*a*^ Percentages were calculated from isolates with interpretable results for each antimicrobial agent; the routine testing panel varied among isolates.

Overall, *Salmonella* isolates remained highly susceptible to carbapenems and some *β*-lactam/*β*-lactamase inhibitor combinations, while relatively high resistance rates were observed for ampicillin, ampicillin/sulbactam, and trimethoprim-sulfamethoxazole.

## DISCUSSION

In this study, we conducted a 5-year retrospective analysis of *Salmonella* spp. isolated from stool specimens of inpatients at a maternal and child healthcare specialty hospital in Shenzhen. From 2021 to 2025, a total of 4,201 stool samples were submitted for culture, among which 176 were positive for *Salmonella* spp.; after duplicate isolates were excluded, 106 non-duplicate isolates were included in the final analysis. Annual positivity and non-duplicate isolation rates differed significantly, and both were highest in 2025. These findings suggest that *Salmonella* spp. remain an important bacterial enteric pathogen requiring continuous attention among stool specimens submitted for suspected intestinal infections in our hospital. Continuous surveillance conducted at Guangzhou Women and Children’s Medical Center from 2016 to 2021 also showed an increasing number of *Salmonella* isolates over time, indicating that the burden of *Salmonella* infection remains clinically relevant in pediatric and maternal–child healthcare settings (23). However, the statistical comparisons evaluated proportions among specimens submitted to the hospital laboratory rather than population-level incidence. Changes in testing indications, inpatient population composition, referral patterns, and clinical sampling practices may have contributed to the observed increase. The 2025 peak should therefore be interpreted as an increase in laboratory-detected cases at our hospital, not as direct evidence of a sustained rise in community incidence.

Regarding population distribution, among pediatric cases in this study, 55 were male and 43 were female, indicating a slight male predominance. The age distribution was strongly concentrated in younger children, particularly those aged 1 year, who accounted for 52.0% of all pediatric cases; the number of detected cases generally decreased with increasing age. The 1-year category represented children aged 12 to <24 months, rather than all children simply described as older than 12 months, because those exactly 12 months of age were included. This age concentration is broadly consistent with previous pediatric studies (12, 24). Plausible contributors include the transition from breast milk or formula to complementary and family foods, increasing mobility and hand-to-mouth behavior, exposure to household food preparation or childcare environments, and continuing maturation of mucosal immunity and the gut microbiota. These explanations are biologically and behaviorally plausible but remain hypotheses because feeding history, food exposure, household transmission, and childcare attendance were not available in our data set. Earlier research has also reported a modest male predominance in pediatric salmonellosis, potentially reflecting a combination of biological, behavioral, and healthcare-seeking factors (24, 25). The higher susceptibility of young children may be associated with the immaturity of the immune system and gut microbiota, increased food exposure after the introduction of complementary feeding, and frequent hand-to-mouth contact. Therefore, in maternal and child healthcare specialty hospitals, clinicians should maintain a high level of vigilance for *Salmonella* infection in young children presenting with diarrhea, fever, or gastrointestinal symptoms, and pathogen testing should be performed early when clinically indicated.

In terms of antimicrobial resistance, the resistance rates of *Salmonella* spp. to ampicillin, ampicillin/sulbactam, trimethoprim/sulfamethoxazole, and ceftriaxone in this study were 76.7%, 63.3%, 46.6%, and 36.9%, respectively, suggesting that the empirical value of these agents for *Salmonella*-associated infections in our hospital has markedly declined. Compared with domestic pediatric studies, our findings show good regional consistency. In surveillance conducted at Hangzhou Children’s Hospital from 2006 to 2021, the resistance rates to ampicillin, ampicillin/sulbactam, trimethoprim/sulfamethoxazole, and ceftriaxone were 71.5%, 51.5%, 36.5%, and 22.4%, respectively (26). Compared with those findings, the resistance levels to ampicillin and ceftriaxone in the present study were generally within a comparable range, whereas resistance to ampicillin/sulbactam and trimethoprim/sulfamethoxazole remained relatively high. These results suggest that high resistance to traditionally used antimicrobial agents among pediatric *Salmonella* isolates persists in both South China and eastern China. A study of foodborne *Salmonella* infection in children in Chenzhou also emphasized that antimicrobial resistance in *Salmonella* has become an important challenge for rational clinical therapy (27). Earlier surveillance of diarrheal patients in Shenzhen likewise documented high ampicillin resistance and extensive multidrug resistance among common *Salmonella* serotypes, providing direct local context for the persistence of resistance (28). Taken together, these findings indicate that ampicillin-based agents or trimethoprim/sulfamethoxazole should no longer be regarded as stable and reliable first-line empirical options, and treatment decisions should rely more heavily on local antimicrobial resistance surveillance data.

Although the present laboratory data set did not contain prescription or pharmacy-consumption data, available Chinese pediatric inpatient data help contextualize regional practice. In a multicenter cohort that included a center in southern China, third-generation cephalosporin-based regimens were used as initial empirical therapy in 53.8% of children with invasive *Salmonella* infection, followed by penicillin-based regimens in 23.8% (14). Thus, third-generation cephalosporins are among the most clinically relevant empirical agents for severe pediatric salmonellosis in Shenzhen and neighboring southern China. The ceftriaxone resistance rate of 36.9% observed in our study is therefore clinically important. However, because individual treatment records and pharmacy-utilization data were not collected, we cannot quantitatively rank antibiotic use within Shenzhen or our institution. Recent studies have suggested that ceftriaxone-resistant *Salmonella* spp. remain a concern, and determinants associated with quinolone resistance are also frequently detected, highlighting the need for simultaneous monitoring of resistance evolution among key therapeutic agents (29). In the present study, the resistance rates to ciprofloxacin and levofloxacin were 12.5% and 14.8%, respectively, which were not the highest rates observed. However, the intermediate susceptibility rates were as high as 50.0% and 64.8%, respectively, indicating that a considerable proportion of isolates had already shown reduced susceptibility. In other words, even if the resistance rates to fluoroquinolones have not yet increased substantially, their actual clinical reliability may already be compromised. For pediatric patients, fluoroquinolones are not routine first-line agents. Therefore, this finding mainly reflects a trend in resistance evolution rather than supporting their earlier routine use in pediatric treatment.

On the other hand, piperacillin/tazobactam and carbapenems retained high *in vitro* activity in this study. The resistance rate to piperacillin/tazobactam was only 3.0%, while the susceptibility rates to imipenem and ertapenem were 97.1% and 98.1%, respectively. These findings are generally consistent with a study of pediatric *Salmonella* infection conducted at Guangdong Women and Children’s Hospital from 2018 to 2023, in which the resistance rates to piperacillin/tazobactam and imipenem were only 2.21% and 0.62%, respectively, suggesting that these agents still maintain good *in vitro* antibacterial activity (30). This indicates that, in severe or complicated infections or in cases of empirical treatment failure, these agents may retain value as therapeutic reserves. However, high susceptibility does not mean that broad-spectrum agents should be routinely moved forward in clinical use. Patients in maternal and child healthcare specialty hospitals are mainly children and pregnant or postpartum women. Therefore, antimicrobial selection must consider not only bacterial coverage but also age-related restrictions, pregnancy safety, and antimicrobial stewardship requirements (31–33). A more appropriate strategy is to develop empirical treatment regimens based on disease severity stratification and local susceptibility data, rather than simply pursuing the broadest possible antimicrobial coverage.

This study has several limitations. First, it was a single-center retrospective study, and the samples were mainly obtained from inpatients at a maternal and child healthcare specialty hospital. Therefore, the results were strongly influenced by the hospital’s patient composition and testing strategy, and extrapolation to general hospitals or other regions should be made with caution. Second, this study was based on laboratory isolate data and lacked more complete clinical information, such as prior antimicrobial exposure, symptom severity, complications, and clinical outcomes. As a result, it was not possible to further analyze infection risk or treatment differences across different clinical subgroups. Third, deduplication was based on the patient identifier and retention of the earliest isolate during the study period. Because sequencing or molecular typing was not performed, genetically distinct recurrent infections in the same patient could have been excluded. Fourth, serotyping, resistance gene detection, and molecular epidemiological analysis were not performed; therefore, the mechanisms underlying ceftriaxone resistance and reduced fluoroquinolone susceptibility could not be explained at the molecular level. Future studies should combine multicenter data with molecular testing to further clarify the epidemiological characteristics and resistance evolution of pediatric *Salmonella* spp. in this region.

Overall, this study shows that *Salmonella* infection in stool specimens from inpatients at Longgang District Maternity and Child Healthcare Hospital of Shenzhen City was mainly concentrated in children aged 1–3 years, particularly those aged 12 to <24 months. The isolates showed high resistance to ampicillin, ampicillin/sulbactam, trimethoprim/sulfamethoxazole, and some cephalosporins, while retaining good activity to piperacillin/tazobactam and carbapenems. Continuous epidemiological and antimicrobial resistance surveillance of *Salmonella* spp. in this region is important for optimizing empirical treatment strategies, improving rational antimicrobial use, and strengthening antimicrobial stewardship in maternal and child healthcare specialty hospitals.

## Supplementary Material

Reviewer comments

## Data Availability

The data analyzed in this study were derived from de-identified hospital laboratory records and are not publicly available. De-identified data may be available from the corresponding author on reasonable request, subject to institutional and ethical requirements.

## SUPPLEMENTAL MATERIAL

OPEN PEER REVIEW reviewer-comments.pdf An accounting of the reviewer comments and feedback.

ASM does not own the copyrights to Supplemental Material that may be linked to, or accessed through, an article. The authors have granted ASM a non-exclusive, world-wide license to publish the Supplemental Material files. Please contact the corresponding author directly for reuse.
