# Supplementary material for: Detection characteristics and antimicrobial resistance profiles of Salmonella spp. isolated from fecal cultures of inpatients at a maternal and child healthcare hospital in Shenzhen: a 5-year retrospective study
Source: Microbiol Spectr. 2026 Oct 8;14(11):e01536-26. doi: 10.1128/spectrum.01536-26 (PMC13729177; doi:10.1128/spectrum.01536-26)
Supplement: Supplementary file 1 [file reviewer-comments.pdf]

ASM | Microbiology Spectrum

Detection Characteristics and Antimicrobial Resistance Profiles of Salmonella spp. Isolated from Fecal Cultures of Inpatients at a Maternal and Child Healthcare Hospital in Shenzhen: A Five-Year Retrospective Study

Tongyan Ding, Shaoxiang Lin, Xiaochun Liu, Shujuan Yang, Futing Liao, qiaoxin Zhang, and Zhenwen Zhou

Corresponding Author(s): Zhenwen Zhou, Longgang District Maternity & Child Healthcare Hospital of Shenzhen City

Review Timeline:

Submission Date:	May 6, 2026
Editorial Decision:	June 26, 2026
Revision Received:	September 4, 2026
Accepted:	September 9, 2026

Editor: Matt Bawn

Reviewer(s): The reviewers have opted to remain anonymous.

Transaction Report:

(Note: With the exception of the correction of typographical or spelling errors that could be a source of ambiguity, letters and reports are not edited. The original formatting of letters and referee reports may not be reflected in this compilation.)

DOI: https://doi.org/10.1128/spectrum.01536-26

Re: Spectrum01536-26 (**Detection Characteristics and Antimicrobial Resistance Profiles of Salmonella spp. Isolated from Fecal Cultures of Inpatients at a Maternal and Child Healthcare Hospital in Shenzhen: A Five-Year Retrospective Study**)

Dear Dr. Zhenwen Zhou:

Thank you for the privilege of reviewing your work. Below you will find my comments, instructions from the Spectrum editorial office, and the reviewer comments.

Please return the manuscript within 60 days; if you cannot complete the modification within this time period, please contact me. If you do not wish to modify the manuscript and prefer to submit it to another journal, notify me immediately so that the manuscript may be formally withdrawn from consideration by Spectrum.

Revision Guidelines
To submit your modified manuscript, log into the submission site at https://spectrum.msubmit.net/cgi-bin/main.plex. Go to Author Tasks and click the appropriate manuscript title to begin. The information you entered when you first submitted the paper will be displayed; update this as necessary. Note the following requirements:

• Upload point-by-point responses to the issues raised by the reviewers in a file named "Response to Reviewers," NOT in your cover letter.
• Upload a compare copy of the manuscript (without figures) as a "Marked-Up Manuscript" file.
• Upload a clean .DOC/.DOCX version of the revised manuscript and remove the previous version.
• Each figure must be uploaded as a separate, editable, high-resolution file (TIFF or EPS preferred), and any multipanel figures must be assembled into one file.
• Any supplemental material intended for posting by ASM should be uploaded with their legends separate from the main manuscript. You can combine all supplemental material into one file (preferred) or split it into a maximum of 10 files with all associated legends included.

For complete guidelines on revision requirements, see our Submission and Review Process webpage. Submission of a paper that does not conform to guidelines may delay acceptance of your manuscript.

Data availability: ASM policy requires that data be available to the public upon online posting of the article, so please verify all links to sequence records, if present, and make sure that each number retrieves the full record of the data. If a new accession number is not linked or a link is broken, provide Spectrum production staff with the correct URL for the record. If the accession numbers for new data are not publicly accessible before the expected online posting of the article, publication may be delayed; please contact production staff (Spectrum@asmusa.org) immediately with the expected release date.

Publication Fees: For information on publication fees and which article types are subject to charges, visit our website. If your manuscript is accepted for publication and any fees apply, you will be contacted separately about payment during the production process; please follow the instructions in that e-mail. Arrangements for payment must be made before your article is published.

ASM Membership: Corresponding authors may join or renew ASM membership to obtain discounts on publication fees. Need to upgrade your membership level? Please contact Customer Service at Service@asmusa.org.

The ASM Journals program strives for constant improvement in our submission and publication process. Please tell us how we can improve your experience by taking this quick Author Survey.

Thank you for submitting your paper to Spectrum.

Sincerely,
Matt Bawn
Editor
Microbiology Spectrum

Reviewer #2 (Comments for the Author):

This manuscript describes an interesting surveillance study on the prevalence of Salmonella and the AMR profile of these isolates. The discission is well-written and informative; however, the methods are unclear and missing details that would allow this study to be duplicated in other regions. Specific comments are below.

Salmonella must be italicized throughout the manuscript.

Please add an age to qualify the work "young" in line 35.

Perhaps the authors could elaborate on the considerations referred to in lines 77 and 78. Also please elaborate on what characteristics of Shenzhen make it so as described in lines 81-82. Is it predominantly the area of the population?

Was there an Institutional Review Board number or protocol available or provided for the authors to conduct the research. The project may have been exempt or this may not have been needed, but please state which is true.

Methods must be added at least in brief in lines 116-118 regarding isolation of Salmonella, including information on what clinical specimens from which these were obtained. Perhaps these are all fecal samples?

Statistical analysis could be added for better data analysis.

How exactly were duplicate isolates determined in Table 1? Were they sequenced?
Can the authors speculate as to what caused the salmonellosis in the 1 year olds? Also please clarify that these cases are all >12 months of age?

The authors should comment on what antibiotics are used most often in this region.

“Detection Characteristics and Antimicrobial Resistance Profiles of Salmonella spp. Isolated from Fecal Cultures of Inpatients at a Maternal and Child Healthcare Hospital in Shenzhen: A Five-Year Retrospective Study.”

The authors have adequately addressed the previous comments and significantly improved the manuscript. The study is well organized, scientifically sound, and provides valuable data regarding antimicrobial resistance patterns of Salmonella spp. in pediatric and maternal healthcare settings.

In my opinion, the manuscript is suitable for publication in its current form.

Recommendation: Accept for publication.

Response to Reviewer #2

Manuscript ID: Spectrum01536-26

Detection Characteristics and Antimicrobial Resistance Profiles of *Salmonella* spp. Isolated from Fecal Cultures of Inpatients at a Maternal and Child Healthcare Hospital in Shenzhen: A Five-Year Retrospective Study

Dear Editor and Reviewer,

We sincerely thank Reviewer #2 for the careful evaluation and constructive suggestions. We have revised the manuscript point by point. Major changes include clarification of the age groups, expansion of the Shenzhen demographic context and antimicrobial-selection considerations, explicit ethical approval information, additional stool-culture and deduplication methods, inferential statistical analysis, discussion of the concentration of cases in the 1-year age group, and contextualization of commonly used antimicrobial classes. The marked-up manuscript shows all additions and revisions in red text. Our detailed responses are provided below.

Page and line numbers below refer to the marked-up revised manuscript.

Comment 1. *Salmonella* must be italicized throughout the manuscript.

Response: We sincerely appreciate the comment. We have italicized *Salmonella* throughout the manuscript, including the Results subsection headings.

Location in marked-up manuscript: Throughout the manuscript; representative examples include Page 1, Lines 1–3 and 16–24; Page 3, Lines 68–71; Page 8, Lines 175–179; and Page 12, Lines 252–256.

Comment 2. Please add an age to qualify the word "young" in line 35.

Response: We agree and have replaced the nonspecific term "young pediatric patients" with an explicit age range. We also report the corresponding numerator, denominator, and percentage, and identify the 1-year category as 12 to <24 months.

Location in marked-up manuscript: Abstract, Page 2, Lines 28–36; Importance, Page 2, Lines 42–46; and age definition, Page 6, Lines 128–131.

Revised text (Abstract): These findings show a concentration of laboratory-detected *Salmonella* among children aged 1 to 3 years, particularly those aged 12 to <24 months, and substantial resistance to several commonly used agents, supporting continued local surveillance and susceptibility-guided antimicrobial use.

Revised text (Importance): The findings show that *Salmonella* spp. isolates were mainly recovered from children aged 1 to 3 years (79.6% of pediatric cases), especially those aged 12 to <24 months, and the isolates exhibited high resistance to several commonly used

antimicrobial agents, including ampicillin, ampicillin/sulbactam, trimethoprim-sulfamethoxazole, and ceftriaxone, while reduced susceptibility to fluoroquinolones was also observed.

Comment 3. Perhaps the authors could elaborate on the considerations referred to in lines 77 and 78. Also please elaborate on what characteristics of Shenzhen make it so as described in lines 81-82. Is it predominantly the area of the population?

Response: Thank you for this helpful suggestion. We clarified that the “considerations” include gestational age, placental transfer, potential fetal or neonatal effects, age- and weight-based dosing, developmental pharmacology, and class-specific precautions or restrictions. We also expanded the Shenzhen description. The intended characteristic was primarily the demographic profile rather than the geographic area: Shenzhen has a large migrant population, a comparatively young age structure, and high residential and cross-district mobility, which create a large and heterogeneous maternal and pediatric service population.

Location in marked-up manuscript: Introduction, Page 4, Lines 74–79 and 82–88.

Revised text (Introduction, paragraph 2): For maternal and child healthcare populations, antimicrobial selection must consider not only pathogen coverage but also gestational age, placental transfer, and potential fetal or neonatal effects in pregnant patients, as well as age- and weight-based dosing, developmental pharmacology, and class-specific precautions or restrictions in children (16).

Revised text (Introduction, paragraph 3): Shenzhen is a rapidly growing megacity with a large migrant population, a comparatively young age structure, and high residential mobility (18-20). Longgang District also receives patients who move across districts or regions for maternal and pediatric care. These demographic and healthcare-seeking characteristics create a large and heterogeneous service population and may influence exposure patterns, testing volume, and the distribution of enteric pathogens observed in hospital-based surveillance.

Comment 4. Was there an Institutional Review Board number or protocol available or provided for the authors to conduct the research? The project may have been exempt or this may not have been needed, but please state which is true.

Response: We agree. The revised manuscript now explicitly states the approving ethics committee, the approval number, and the waiver of informed consent. The study was not described as exempt; it received ethics approval, and informed consent was waived because the analysis used de-identified routine laboratory data without additional sampling or intervention.

Location in marked-up manuscript: Materials and Methods, Ethics statement, Page 8, Lines 167–173.

Revised text (Materials and Methods, Ethics statement): This study was approved by the Research Project Ethics Committee of Longgang District Maternity and Child Healthcare Hospital of Shenzhen City (approval no. LGFYKYXMLL-2026-2). Because this was a retrospective analysis based on de-identified routine laboratory data and involved no identifiable private information, additional sample collection, or clinical intervention, the requirement for informed consent was waived by the ethics committee.

Comment 5. Methods must be added at least in brief in lines 116-118 regarding isolation of *Salmonella*, including information on what clinical specimens from which these were obtained. Perhaps these are all fecal samples?

Response: We agree. We clarified that every included clinical specimen was a fecal specimen and added the main culture workflow: selective/differential enteric culture, enrichment when indicated by the routine stool-culture protocol, aerobic incubation, purification of suspected colonies, automated identification and susceptibility testing, and MALDI-TOF MS confirmation when required.

Location in marked-up manuscript: Study design and isolate source, Pages 5–6, Lines 111–116; Isolation, identification, and antimicrobial susceptibility testing, Pages 6–7, Lines 132–147.

Revised text (Materials and Methods, Study design and isolate source): All clinical specimens included in the analysis were fecal specimens.

Revised text (Materials and Methods, Isolation, identification, and antimicrobial susceptibility testing): Fecal specimens were processed according to the routine stool-culture standard operating procedure of the clinical microbiology laboratory. Specimens were inoculated onto selective and differential enteric culture media, with enrichment performed when indicated by the routine protocol, and incubated aerobically at 35 to 37 degrees C for 18 to 48 h. Colonies with morphology suggestive of *Salmonella* were subcultured to obtain pure growth before identification and susceptibility testing.

Comment 6. Statistical analysis could be added for better data analysis.

Response: We agree. We added Pearson chi-square tests comparing the annual positivity rates and annual non-duplicate isolation rates across 2021-2025. Both overall comparisons were statistically significant. We did not perform inferential comparisons for age or sex because the corresponding age- and sex-specific denominators for all submitted specimens were unavailable. We also clarified that these tests assess laboratory detection proportions rather than population incidence.

Location in marked-up manuscript: Data collection and statistical analysis, Pages 7–8, Lines 153–166; Results, Page 9, Lines 185–188.

Revised text (Materials and Methods, Data collection and statistical analysis): Pearson chi-square tests were used to compare the annual positivity rates and annual non-duplicate isolation rates across the five study years. Age and sex distributions were summarized descriptively because age- and sex-specific denominators for all submitted specimens were unavailable. All tests were two-sided, and $P < 0.05$ was considered statistically significant.

Revised text (Results, Detection of Salmonella spp. among fecal culture isolates): The annual positivity rate differed significantly across the five years (Pearson chi-square = 80.52, df = 4, $P < 0.001$), as did the annual non-duplicate isolation rate (Pearson chi-square = 35.97, df = 4, $P < 0.001$). Both rates were highest in 2025.

Comment 7. How exactly were duplicate isolates determined in Table 1? Were they sequenced?

Response: Thank you for requesting clarification. Duplicate removal was based on the hospital patient identifier in the laboratory information system. If a patient had more than one *Salmonella*-positive fecal culture during the full study period, only the earliest isolate was retained, regardless of the interval or susceptibility profile. The isolates were not sequenced or molecularly typed for deduplication. We have added this definition to the Methods and Table 1 note and have acknowledged that the approach may exclude genetically distinct recurrent infections in the same patient.

Location in marked-up manuscript: Definitions and deduplication, Page 6, Lines 118–124; Table 1 note, Page 9, Lines 191–193; Discussion (Limitations), Pages 17–18, Lines 364–367.

Revised text (Materials and Methods, Definitions and deduplication): If a patient had more than one *Salmonella*-positive fecal culture during the entire study period, only the earliest isolate was retained, irrespective of the interval between cultures or any difference in antimicrobial susceptibility profile. Deduplication was therefore patient based; whole-genome sequencing, targeted sequencing, and other molecular typing methods were not used to determine whether isolates were genetically identical.

Revised text (Discussion, Limitations): Because sequencing or molecular typing was not performed, genetically distinct recurrent infections in the same patient could have been excluded.

Comment 8. Can the authors speculate as to what caused the salmonellosis in the 1 year olds? Also please clarify that these cases are all >12 months of age?

Response: We have clarified the age definition and expanded the Discussion. Age was grouped by completed year: the 1-year category includes patients aged at least 12 months but younger than 24 months. It is therefore not strictly “>12 months,” because children exactly 12 months old are included. No pediatric case was recorded in the <12-month (age 0) category. We added plausible explanations for the concentration in this age group, including complementary/family-food exposure, increasing mobility and hand-to-mouth behavior,

household or childcare exposure, and continued maturation of mucosal immunity and the gut microbiota. We explicitly state that these are hypotheses because the study lacked feeding and exposure data.

Location in marked-up manuscript: Definitions and deduplication, Page 6, Lines 128–131; Results (age distribution), Page 10, Lines 209–214; Discussion, Pages 13–14, Lines 271–284.

Revised text (Materials and Methods, Definitions and deduplication): Patient age was calculated from date of birth to specimen-collection date and categorized by completed year of age. Accordingly, the 1-year age group comprised patients aged at least 12 months but younger than 24 months.

Revised text (Discussion, age-distribution paragraph): Plausible contributors include the transition from breast milk or formula to complementary and family foods, increasing mobility and hand-to-mouth behavior, exposure to household food preparation or childcare environments, and continuing maturation of mucosal immunity and the gut microbiota. These explanations are biologically and behaviorally plausible but remain hypotheses because feeding history, food exposure, household transmission, and childcare attendance were not available in our dataset.

Comment 9. The authors should comment on what antibiotics are used most often in this region.

Response: We agree and have added regional clinical context while avoiding an unsupported quantitative claim about our hospital. A Chinese multicenter pediatric cohort that included a southern China center reported that third-generation cephalosporin-based regimens were the most common initial empirical therapy (53.8%), followed by penicillin-based regimens (23.8%). We therefore explain why ceftriaxone resistance is especially relevant for Shenzhen and neighboring southern China. We also state clearly that our laboratory-only dataset did not include individual prescriptions or pharmacy-consumption data and therefore cannot quantitatively rank antibiotic use within Shenzhen or our institution.

Location in marked-up manuscript: Discussion, Pages 15–16, Lines 316–326.

Revised text (Discussion, antimicrobial-use paragraph): In a multicenter cohort that included a center in southern China, third-generation cephalosporin-based regimens were used as initial empirical therapy in 53.8% of children with invasive *Salmonella* infection, followed by penicillin-based regimens in 23.8% (14). Thus, third-generation cephalosporins are among the most clinically relevant empirical agents for severe pediatric salmonellosis in Shenzhen and neighboring southern China. The ceftriaxone resistance rate of 36.9% observed in our study is therefore clinically important. However, because individual treatment records and pharmacy-utilization data were not collected, we cannot quantitatively rank antibiotic use within Shenzhen or our institution.

We again thank the reviewer for these comments, which helped us improve the clarity, reproducibility, and clinical interpretation of the manuscript.

Re: Spectrum01536-26R1 (**Detection Characteristics and Antimicrobial Resistance Profiles of Salmonella spp. Isolated from Fecal Cultures of Inpatients at a Maternal and Child Healthcare Hospital in Shenzhen: A Five-Year Retrospective Study**)

Dear Dr. Zhenwen Zhou:

Your manuscript has been accepted, and I am forwarding it to the ASM production staff for publication. Your paper will first be checked to make sure all elements meet the technical requirements. ASM staff will contact you if anything needs to be revised before copyediting and production can begin. Otherwise, you will be notified when your proofs are ready to be viewed.

Data Availability: ASM policy requires that data be available to the public upon online posting of the article, so please verify all links to sequence records, if present, and make sure that each number retrieves the full record of the data. If a new accession number is not linked or a link is broken, provide production staff with the correct URL for the record. If the accession numbers for new data are not publicly accessible before the expected online posting of the article, publication may be delayed; please contact ASM production staff immediately with the expected release date.

Publication Fees: For information on publication fees and which article types have charges, please visit our website. We have partnered with Copyright Clearance Center (CCC) to collect author charges. If fees apply to your paper, you will receive a message from no-reply@copyright.com with further instructions. For questions related to paying charges through RightsLink, please contact CCC at ASM_Support@copyright.com or toll free at +1-877-622-5543. CCC makes every attempt to respond to all emails within 24 hours.

ASM Membership: Corresponding authors may join or renew ASM membership to obtain discounts on publication fees. Need to upgrade your membership level? Please contact Customer Service at Service@asmusa.org.

Embargo Policy: A press release may be issued as soon as the manuscript is posted on the Spectrum Latest Articles webpage. The corresponding author will receive an email with the subject line "ASM Journals Author Services Notification" when the article is available online.

Cover Image Submissions: If you would like to submit a potential Cover Image, please email a file and a short legend to spectrum@asmusa.org. Please note that we can only consider images that (i) the authors created or own and (ii) have not been previously published. By submitting, you agree that the image can be used under the same terms as the published article. Image File requirements: TIF/EPS, 7.5 inches wide by 8.25 inches tall (at least 2,250 pixels wide by 2,475 pixels tall), minimum 300 dpi resolution (600 dpi preferred), RGB, and no figure elements, e.g., arrows or panel labels. The legend should be a short description of the image, 1-2 sentences recommended. Please use the ASM Journals Cover Image Sizing Tool (https://journals.asm.org/pb-assets/html/asm-cover-sizing-tool.html) to ensure that your proposed cover image meets our size requirements.

The ASM Journals program strives for constant improvement in our submission and publication process. Please tell us how we can improve your experience by taking this quick Author Survey.

Thank you for submitting your paper to Spectrum.

Sincerely,
Matt Bawn
Editor
Microbiology Spectrum
